# Supplementary material for: Polarization and trust in the evolution of vaccine discourse on Twitter during COVID-19
Source: PLoS One. 2022 Dec 14;17(12):e0277292. doi: 10.1371/journal.pone.0277292 (PMC9749990; doi:10.1371/journal.pone.0277292)
Supplement: S1 Appendix — This includes data collection considerations (incl. hashtags), analysis of bots in the dataset, biases in data, classification tasks, and structural break analyses. (ZIP) [file pone.0277292.s001.zip › Supplementary Material.pdf]

Supporting Information for "Polarization and trust in the evolution of vaccine discourse on Twitter during COVID-19"

Data Collection

We queried the Twitter Streaming API with a series of vaccination-related keywords, hashtags, and short expressions between December 2019 and June 2020. The following pre-registered vaccination-related keywords, hashtags and short expressions were used: *'vax', 'vaxxed', 'vaccine', 'vaccination', 'vaccinations', 'vaxsafety', 'vaccineswork', 'vaccines work', 'vaccines revealed', 'vaccinesrevealed', 'novax', 'no vax', 'no-vax', 'antivax', 'anti-vax', 'anti vax', 'immunisation', 'Vaccin', 'Vaccinaties', 'vaccinatiezorg', 'vaccine injury', 'vax injury','vaccinatieschade', '#vax', '#vaxxed', '#vaccine', '#vaccination', '#vaccinations', '#vaxsafety', '#vaccineswork', #vaccinesrevealed', '#novax', '#antivax', '#immunisation', '#Vaccin', '#Vaccinaties', '#vaccinatiezorg', '#vaccinatieschade', '#nvkp', '#rvp', '#rijksvaccinatieprogramma', '#vaccineinjury', '#vaxinjury', '#anti-vax'.* The choice of words was done following similar literature on vaccination discourse on Twitter [1], and with the goal of trying to capture a wide spectrum vaccine related attitudes.

Bot detection and analysis

As with any social network, Twitter is not immune to interference by bots – automated or semi-automated software designed to create posts and engage with users/tweets. Originally used for less-nefarious purposes, such as providing scheduled feeds [2], bots on Twitter are now synonymous with disinformation campaigns. There are various heuristics used to detect bots – both the benign and the malevolent kinds – and the area of bot detection has been ongoing since ca. 2010; key literature on the topic can be found in [2, 3] and more recently [4].
For completeness, we attempt to identify the proportion of bot-generated retweets coming from each community in our analysis, using readily-available user metadata. We used the heuristic of median time lag per user (*MTL*). This is defined on a per-user basis as $MTL_{user} = median_{i=1}^{|R_{user}|}(rt_i - op_i)$; where R_{user} = set of all retweets by *user*, op_i = time of an original post i, and rt_i = time that post i was actually retweeted by the *user* in question. Any user with a *MTL* of less than 10.0 seconds [4] is indicative of bot activity. In other words, the median of all retweets by the user is within less than ten seconds of their original posts being visible on Twitter, which is highly impractical for a human user to achieve.
We identified 1087 accounts with metadata exhibiting an MTL < 10; only 499 of them belong to the top five largest communities. Relative to the more than 300K users within those communities, this figure is small enough to isolate, but we deliberately chose to include them in our analysis.

Data Biases

In regards to data bias, we refer to e.g. [2], who was one of the pioneers of Twitter Streaming API data collection. In this work, the author explains that Twitter employs a random sampling method. Furthermore, we performed several power law analyses explained in the following section to establish the representativeness of our dataset.

To test for biases in our data, we conducted a power law analysis. Both weighted and unweighted degree versions of the network follow a statistically significant ($p = 0.0127$ and 0.0092 respectively) power law distribution when compared with the lognormal distribution [5]. On the one hand, this means that the retweet distribution is approximately a power law. These are recurrent in social media [2] and in particular for retweet networks [6, 7]. Accordingly, [6] propose modeling retweet patterns using power laws and are able to develop a model that successfully reproduces empirical data based on their assumptions. On the other hand, 80% of nodes had fewer than five neighbors (retweeters) and only 5% of nodes were retweeted by more than 20 distinct nodes, which shows that a small minority was highly influential. Our observations are consistent with the literature, which suggests that our vaccine-focused subgraph is representative of extant research and not biased.

Classification Task

For the classification task we used the labeling obtained from community detection to create our training and testing sets. For each user in our network we generated a document containing the plain text of tweets they authored. Tweet text was then pre-processed in standard fashion (remove non alpha-numeric, tolower, etc.). Resultantly, each user has a tweet corpus and a community label. Training and testing sets were done normalizing by the smallest community (Antivaxxers with $\sim$ 30K authors), so that there is an equal number of authors for all clusters.

For the classifiers, we used two different data representations, corresponding to two different types of classifiers. On the one hand, for more classical machine learning techniques (Scikit-Learn logistic regression, and random forests) we used the bag of words representation. Here, word (including emoji and hashtag) tokens are features and each author's document gets a score corresponding to the occurrences of that token in the document. On the other hand, for newer deep learning techniques (Tensorflow GRUs and LSTMs) we used a sequential representation, since some of those techniques can detect sequential patterns. Each document is now a sequence, where each word token is encoded as a number. Later, in learning, those numbers are embedded into a vector, so that each document is effectively a matrix.

The task was done in a multi-class setup (one class per community) and a binary setup (Antivaxxers or others). For evaluation, we chose accuracy. Results reported below were obtained after hyper-parameter tuning to obtain maximum performance.

On the one hand, we considered a multi-class setup with a label for each of the five communities we identified. On the other hand, a binary classification task between the Antivaxxer community and the others. Furthermore, we used both standard machine learning techniques like (multinomial) Logistic Regresion and Random Forests, and well established deep learning techniques like Long short-term memory (LSTM) and Gated recurrent unit (GRU) networks. The results for the classification task are shown in Table 1 below.

As Table 1 shows, all classification tasks greatly outperformed random classification. Data was normalized so as to have an equal amount of users for each community. This means that in the multi-class task (5 labels) random classification has an expected accuracy of 20%, and in the binary task of 50%. Our classifiers performed above 50% in multi-class and above 70% in binary classification.[1]

We performed the community detection on a network obtained by *retweet* behavior, yet part of the project here is to study vaccine *discourse*. Hence it is reasonable to ask: How much is retweet behavior correlated with linguistic behavior and How distinctive is

[1]For the binary classification logistic regression and random forests had an Area Under the Curve (AUC) of 0.81, while had 0.84 (GRU) and 0.85 (LSTM).

Classifier	Accuracy	Task Type	Random Accuracy
Logistic Regression	0.52	Multi-Class	0.20
Random Forest	0.51	Multi-Class	0.20
GRU	0.51	Multi-Class	0.20
LSTM	0.50	Multi-Class	0.20
Logistic Regression	0.73	Binary	0.5
Random Forest	0.74	Binary	0.5
GRU	0.76	Binary	0.5
LSTM	0.77	Binary	0.5

Table 1. Classification task results

the language use of each of the communities we identified? The current results suggest that language use is a reliable classification base for many of the groups.

Structural break

Following the time series analysis methodology described earlier, we also tested the hypothesis that the pandemic declaration changed how groups related by performing a Quandt Likelihood ratio (QLR) for a structural break, using aggregated influence across all groups and performing a Chow test at each point of a 15% trimmed timeseries and looking for the highest F-value greater than the critical threshold. Turning to the change over the course of the pandemic, the QLR test showed evidence of a significant structural break ($F = 56.8, q = 5, \alpha = 0.01, QLR = 4.53$) on March 16th, 2020. As Fig 1 shows, this occurs 5 days after the WHO declaration.

Fig 1. QLR test and public health measures

References

1. Shah Z, Surian D, Dyda A, Coiera E, Mandl K, Dunn A. Automatically appraising the credibility of vaccine-related web pages shared on social media: A Twitter surveillance study. Journal of Medical Internet Research. 2019;21(11).

2. Cheong M. Inferring social behavior and interaction on Twitter by combining metadata about users & messages; 2013.

3. Alothali E, Zaki N, Mohamed EA, Alashwal H. Detecting Social Bots on Twitter: A Literature Review. In: 2018 International Conference on Innovations in Information Technology (IIT); 2018. p. 175–180. Available from: `http://dx.doi.org/10.1109/INNOVATIONS.2018.8605995`.

4. Smith ST, Kao EK, Mackin ED, Shah DC, Simek O, Rubin DB. Automatic detection of influential actors in disinformation networks. Proc Natl Acad Sci U S A. 2021;118(4).

5. Alstott J, Bullmore E, Plenz D. Powerlaw: A Python Package for Analysis of Heavy-tailed Distributions. PLOS ONE. 2014;9(4):e95816.

6. Lu Y, Zhang P, Cao YN, Hu Y, Guo L. On the Frequency Distribution of Retweets. Procedia Computer Science. 2014;31:747–753. doi:10.1016/j.procs.2014.05.323.

7. Bild DR, Liu Y, Dick RP, Mao ZM, Wallach DS. Aggregate Characterization of User Behavior in Twitter and Analysis of the Retweet Graph. ACM Trans Internet Technol. 2015;15(1). doi:10.1145/2700060.
