# Supplementary material for: Polarization and trust in the evolution of vaccine discourse on Twitter during COVID-19
Source: PLoS One. 2022 Dec 14;17(12):e0277292. doi: 10.1371/journal.pone.0277292 (PMC9749990; doi:10.1371/journal.pone.0277292)
Supplement: Attachment — Submitted filename: Response to Reviewers - PLOS ONE.pdf [file pone.0277292.s002.pdf]

Dr Ignacio Ojea Quintana and coauthors
Australian National University
Canberra
ACT 2600
Australia

June 4th, 2022.

Re: Response to Reviewers, PLOS ONE / PONE-D-21-40016

Dear Professor Zhu on behalf of the Editors and Reviewers,

We thank you, the Editorial Board, and the panel of Reviewers for your generous time in the review process of our paper and for providing us with helpful comments on our manuscript, “Polarization and trust in the evolution of vaccine discourse on Twitter during COVID-19”.

On the following pages, please find our detailed responses to address the comments raised by the reviewer panel.

Thank you for your time and consideration.

Kind regards,

Dr Ignacio Ojea Quintana

on behalf of all coauthors
(Ignacio Ojea Quintana, Ritsaart Reimann, Marc Cheong, Mark Alfano, Colin Klein)

Encl. (Responses to the Review Report)

**Responses to the Review Report**

**Please find our responses to each point (in italics) within the corresponding boxes below each point.**

*Journal Requirements:*

*When submitting your revision, we need you to address these additional requirements.*

*1. Please ensure that your manuscript meets PLOS ONE's style requirements, including those for file naming. The PLOS ONE style templates can be found at https://journals.plos.org/plosone/s/file?id=wjVg/PLOSOne_formatting_sample_main_body.pdf*

*and*

*https://journals.plos.org/plosone/s/file?id=ba62/PLOSOne_formatting_sample_title_authors_affiliations.pdf*

Thank you to the Editor for the kind reminder. We have ensured that the default formatting parameters in LaTeX (margins, alignment, spacing, etc) are in accordance with the PLOS ONE LaTeX template (https://journals.plos.org/plosone/s/file?id=SyJ3/plos-latex-template.zip ).

Specifically, Tables 1 and 2 have been formatted to comply with PLOS ONE's table styles.

*2. Please ensure that you refer to Figure 6 in your text as, if accepted, production will need this reference to link the reader to the figure. (Ignacio)*

Figure 6, as well as all other figures in the paper are now referenced in the main body of the text. Furthermore, all figures were normalized using the automated figure assessment PACE.

*3. Please include captions for your Supporting Information files at the end of your manuscript, and update any in-text citations to match accordingly. Please see our Supporting Information guidelines for more information: http://journals.plos.org/plosone/s/supporting-information*

We thank the Editor for this guideline. We have updated the nomenclature for the S1 Appendix; updated the S1 Appendix filename [S1_Appendix.pdf] as required; added a subtitle to the S1 Appendix; and ensured all in text citations are literally "S1 Appendix". Finally we have added the 'Caption' section before the References.

*4. Please review your reference list to ensure that it is complete and correct. If you have cited papers that have been retracted, please include the rationale for doing so in the manuscript text, or remove these references and replace them with relevant current*

*references. Any changes to the reference list should be mentioned in the rebuttal letter that accompanies your revised manuscript. If you need to cite a retracted article, indicate the article's retracted status in the References list and also include a citation and full reference for the retraction notice.*

We thank the Editor for the kind reminder. Our team has gone through the references and double checked everything. Nothing we cite has been retracted, and citations are complete.

*Additional Editor Comments (if provided):*

We thank the Editor for compiling the helpful suggestions on PLOS ONE's formatting requirements as above.

***Comments to the Author***

*1. Is the manuscript technically sound, and do the data support the conclusions?*

*The manuscript must describe a technically sound piece of scientific research with data that supports the conclusions. Experiments must have been conducted rigorously, with appropriate controls, replication, and sample sizes. The conclusions must be drawn appropriately based on the data presented.*

*Reviewer #1: Yes Reviewer #2: Yes Reviewer #3: Yes Reviewer #4: Yes*

*2. Has the statistical analysis been performed appropriately and rigorously?*

*Reviewer #1: Yes Reviewer #2: Yes Reviewer #3: Yes Reviewer #4: Yes*

*3. Have the authors made all data underlying the findings in their manuscript fully available?*

*The PLOS Data policy requires authors to make all data underlying the findings described in their manuscript fully available without restriction, with rare exception (please refer to the Data Availability Statement in the manuscript PDF file). The data should be provided as part of the manuscript or its supporting information, or deposited to a public repository. For example, in addition to summary statistics, the data points behind means, medians and variance measures should be available. If there are restrictions on publicly sharing data—e.g. participant privacy or use of data from a third party—those must be specified.*

*Reviewer #1: Yes Reviewer #2: Yes Reviewer #3: Yes Reviewer #4: Yes*

*4. Is the manuscript presented in an intelligible fashion and written in standard English?*

*PLOS ONE does not copyedit accepted manuscripts, so the language in submitted articles must be clear, correct, and unambiguous. Any typographical or grammatical errors should be corrected at revision, so please note any specific errors here.*

*Reviewer #1: Yes Reviewer #2: Yes Reviewer #3: Yes Reviewer #4: Yes*

We thank all Reviewers for their kind and thorough review of our research, and for confirming the core requirements 1-4 above are met.

5. ***Review Comments to the Author***

*Please use the space provided to explain your answers to the questions above. You may also include additional comments for the author, including concerns about dual publication, research ethics, or publication ethics. (Please upload your review as an attachment if it exceeds 20,000 characters)*

*Reviewer #1: The article has been written meticulously and addresses an important public health issue of developed and developing nations alike using an innovative approach. In my opinion all the findings and interpretations are scientifically sound as presented in the paper.*

We thank Reviewer #1 for their positive review of our paper.

*Reviewer #2: The manuscript is written in very prominent contemporary debate. Scientifically written in logical flows. "We have argued that this realignment shows that the dynamics of online discourse are driven by social connection and trust, rather than by underlying static values." Agreed.*

We thank Reviewer #2 for their positive review of our paper.

*Reviewer #3: The authors describe an analysis of the debate about COVID-19 on Twitter over the first five months of the pandemic. The final aim is to study English-language discourse around vaccines, vaccination-related engagement and discourse, and the causes of the changes in engagement. They describe the data collected, their approach, and the observations derived from the analyses. The authors focus their research on the top five communities they extracted (as they represent most of the English-language data they collected), summarising their statistics, and focusing all of their analyses on these groups. The authors provide detailed descriptions of the outcomes of their study by answering their research questions and properly discussing the limitations of their approach.*

*The article is well organised, and the outcomes are discussed thoroughly. I advise providing additional context and explanations for the following points.*

We thank Reviewer #3 for their positive review of our paper, their succinct summary of our key points, and for their helpful feedback. Please find below our improvements and replies to the comments, itemized for the Editor’s convenience.

*1. In the data collection part, when mentioning "...a series of vaccination-related keywords, hashtags, and short expressions…", it would be good to have a few examples of such words/expressions (even if they are included in the Appendix) with a small discussion on how and why such words were chosen (maybe you could pick the most relevant and/or complex ones).*

The main file now contains a footnote with a few examples of the keywords, and the Appendix includes the complete list with a brief discussion of the choice of words. This was done following similar literature on vaccination discourse on Twitter (1), and with the goal of trying to capture a wide spectrum vaccine related attitudes.

*2. The authors discarded "Users that only retweeted but never authored an original tweet" while keeping "...a very small number of bots". Both users with retweets only and bots may play a signal-boosting function. Discussing the reasons behind the decision of keeping one category while discarding the other may improve the soundness of the article. Moreover, how different would the network be without bots' data? Do bots really change the shape of the network or the interactions between the communities? (Ignacio, Marc, Rits, Colin)*

Two points are worth noting. First, the overall small number of bots (499 out of ~380K total users - See S1 Appendix for details) gives us confidence that the linguistic results will not be seriously skewed by any oddity in bot content. This is now noted in the main text.

Second, we note that we have treated bots and non-bot users uniformly, in the sense that bots are themselves only included if they make original tweets as well. These are likely to be unusual bots (or, perhaps, hybrid human/bot accounts), and the fact that they both retweet and make original tweets is reason to believe that they would play the same functional role in bringing together communities as would a similarly situated human user. This was one reason why we opted to keep the small number of detected bots in the analysis. We have also noted this in the main text.

*3. The authors considered only the top five communities, mainly because they share content using the English language while representing 80% of the nodes and 90% of the tweets. How and why did the authors choose only the top five? What is the choice criterion? Was it only because of the language used within the identified communities?*

The choice to focus on the top 5 communities was informed by several criteria.
First, since these top 5 communities covered 80% of nodes and 90% of retweets, we take them to be representative of the phenomena we are trying to capture.
Second, much of the rest of the nodes were non-English speakers (mostly French), and the dictionary based natural language processing tools we used cannot be meaningfully applied to them.
Third, besides the top five and a French speaking community, the rest of the communities could not be given a straightforward qualitative description.
Fourth and finally, we do believe that our result is up to some degree biased with respect to the USA and UK, and we were able to identify small subcommunities with more global representation. In particular, a subcommunity of African users (within the Unorthodox), and a subcommunity related to the Indian media ecosystem (not included). We do think there is room for broader study of vaccination discourse with a more global perspective, and we have been already having collaborations in this regard. But bringing this conversation into the paper

would mean obscuring the overall picture it is offering.

*4. The authors state "Communities beyond the top five also tended to cluster around non-English-language accounts, which limit the utility of our dictionary-based tools". Did the authors discard all the non-English content the top five communities shared? I advise discussing such a point and carrying out a quick analysis of the percentages of English content VS non-English for the top five (or even 10) groups to cover such an aspect, also providing further support for the discussion about the choice of the top five communities.*

The preprocessing of the linguistic content included removing non-English words and characters; and this is now explained in the main body of the paper. Furthermore, the fact that our linguistic tools are calibrated to pick up *only* on english terms effectively means that all non-english language was excluded from our analyses. For further support as concerns focusing only on the top 5 communities, see previous point.

*Furthermore, the authors mention the Appendix many different times within the text, although no Appendix is provided within the article.*

Thanks to Reviewer #4 for this heads-up. We have ensured that the S1 Appendix is captioned accordingly before the References list, compliant with the PLOS author guidelines (https://journals.plos.org/plosone/s/supporting-information#loc-item-description ) - see also item (3) under [Journal Requirements] above.

*Reviewer #4: 1. The article is very clear and well defined. But still some corrections are presents.*

We thank Reviewer #4 for their positive review of our paper and for their helpful feedback. Please find below our improvements and replies to the comments, itemized for the Editor's convenience.

*2. Authors need to focus the novelty in abstract section and expected output with actual output.*

We thank Reviewer #4 for this suggestion, since it helped us improve the abstract. We now emphasize there the research questions that guided our study, and the results obtained. It now reads:
"Trust in vaccination is eroding, and attitudes about vaccination have become more polarized. This is an observational study of Twitter analyzing the impact that COVID-19 had on vaccine discourse over the first 5 months of the pandemic by identifying the actors, how their language changed, and what can explain this change.
First, we find that authors cluster into several large, interpretable groups, and that the discourse was greatly affected by American partisan politics. Over the course of our study, both Republicans and Democrats entered the vaccine conversation in large numbers, forming coalitions with Antivaxxers and public health organizations, respectively. After the pandemic was officially declared, the interactions between these groups increased. Second, we show how the moral and non-moral language used by the various communities converged in interesting and informative ways. Finally, in order to explain this, vector autoregression analysis indicates that public health measures are likely part of what drove this convergence. Furthermore, building in our linguistic results, we suggest that this was ultimately driven by a trust-first dynamic of political engagement.``

*3. introduction section is too short to intro the proposed work motto.*

The introduction is now expanded and clarified. It now lays out more clearly how we proceeded to answer each of the guiding questions, a summary of the results, and a description of the structure of the paper.

*4. Result section is very organized but images are unclear. Need to upgrade with high resolution images.*

We have now enhanced the resolution for most of the figures and used the automated figure assessment software PACE to check for their compliance to standards, as it is suggested in the PLOS One Figure requirements. Although we hope that this satisfies the reviewer, we are happy to render them again.

*5. Try to use some recent papers as related work (2020-2022). (Rits)*

We have actioned this point by adding ~20 citations from 2022 and beyond, for completeness. Thank you to the Reviewer #4 for this suggestion.

*6. There are some grammatical corrections, please recheck and correct.*

We thank Reviewer #4 for the kind reminder. This should be in order now: we have checked the grammar of the paper and ensured the manuscript is compliant with the APA style guide on writing (e.g. phraseology and standardisation of terms).
