# Supplementary material for: Polarization and trust in the evolution of vaccine discourse on Twitter during COVID-19
Source: PLoS One. 2022 Dec 14;17(12):e0277292. doi: 10.1371/journal.pone.0277292 (PMC9749990; doi:10.1371/journal.pone.0277292)
Supplement: Attachment — Submitted filename: Reviews 2nd R&R.pdf [file pone.0277292.s003.pdf]

Dr Ignacio Ojea Quintana and coauthors

Australian National University
Canberra
ACT 2600
Australia

August 10th, 2022.

Re: Response to Reviewers, PLOS ONE / PONE-D-21-40016

Dear Hanna Landenmark and Tingshao Zhu, on behalf of the Editors and Reviewers,

We thank you, the Editorial Board, and the panel of Reviewers for your generous time in the review process of our paper and for providing us with helpful comments on our manuscript, “Polarization and trust in the evolution of vaccine discourse on Twitter during COVID-19”.

On the following pages, please find our detailed responses to address the comments raised by the reviewer panel.

Thank you for your time and consideration.

Kind regards,

Dr Ignacio Ojea Quintana

on behalf of all coauthors
(Ignacio Ojea Quintana, Ritsaart Reimann, Marc Cheong, Mark Alfano, Colin Klein)

Encl. (Responses to the Review Report)

Responses to the Review Report

Reviewer #3:

The authors presented a study analysing the impact of COVID-19 on the vaccine discourse over the first 5 months of pandemic on Twitter. They clearly stated the research questions they address and provide detailed analyses covering such questions. The authors properly describe the methods, data, and procedures employed in the process.

We thank the reviewer for their charitable comments.

Reviewer #4:

Authors are successfully addressed all of the comments from reviewer. No further corrections are identified. (sic.)

> We are glad to have addressed the comments, and we thank the reviewer for them since they genuinely improved the essay.

Reviewer #5:

The topic presented by the authors is very interesting and timely. However, there are several major issues that need to be addressed.

We thank the reviewer for their comments.<br>In the next paragraphs we will address them one by one.

1. The abstract is very vague and not very clear. Please revise in a way the reader knows exactly what to expect from the paper.

The abstract was modified to improve clarity. It now reads:<br>"Trust in vaccination is eroding, and attitudes about vaccination have become more polarized. This is an observational study of Twitter analyzing the impact that COVID-19 had on vaccine discourse. We identify the actors, the language they use,how their language changed, and what can explain this change.<br>First, we find that authors cluster into several large, interpretable groups, and that the discourse was greatly affected by American partisan politics. Over the course of our study, both Republicans and Democrats entered the vaccine conversation in large numbers, forming coalitions with Antivaxxers and public health organizations, respectively. After the pandemic was officially declared, the interactions between these groups increased. Second, we show how the moral and non-moral language used by the various communities converged in interesting and informative ways. Finally, vector autoregression analysis indicates that differential responses to public health measures are likely part of what drove this convergence. Taken together, our results suggest that polarization around vaccination discourse in the context of COVID-19 was ultimately driven by a trust-first dynamic of political engagement."<br><br>The abstract states our three main contributions: (i) an observational study of which communities were identified, (ii) an analysis of how their language changed, and (iii) an explanatory hypothesis using vector autoregression, complemented with another hypothesis about what drove the dynamics.<br><br>In the body of the paper we also made an effort to be more precise in the description of the techniques used and the numerical results. But since all other reviewers found the original abstract sufficiently clear, we do not want to make substantial changes to the original version.

2. I like highlighting RQ1 - RQ3 on page 2. However, I suggest to be more specific.

RQ1. Which groups are most important in the English-language discourse around vaccines on Twitter? I do not think that is what the authors do. Instead, the focus is on specific countries using the English language. Which countries are these? Add to RQ1!

RQ2. How did vaccination-related engagement and discourse change over the first five months of the pandemic? Add years, ie. 02/2000 - 07/2000 or similar.

RQ3. What social forces might help explain observed changes in engagement? should be

RQ3. What social forces might help in explaining observed changes in engagement?

> We thank the reviewer for asking us to sharpen our research questions. With respect to RQ2 and RQ3, the suggested changes have been incorporated. With respect to RQ1, we have added some comments to clarify that we *did not* limit data collection to the United States nor any other territory, and that the prevalence of specific regions within our dataset is an artifact of the interaction between our methodology and the distribution of Twitter's user-base. In particular, we point out that the prevalence of U.S-based users within our analysis is partly due to our focus on English-language discourse; partly due to the fact that the vast majority of Twitters English speaking user-base is located in the United States; and partly a reflection of the extent to which American discourse defines global online discussions, at least when those discussions are carried out in English.

In general, the language needs to be improved of the paper. In its current form it is very poor language at many places and I cannot list all of them.

As explained in the sections coming (just another example of poor language).

> In the next few responses we explain thow we clarified the language used and the structure of the essay in a way that is clearly tied with the observations we made.

3.To identify different communities, we used Gephi's implementation of the Louvain 110 modularity algorithm developed by [22] This is unclear. Please discuss community/module detection algorithms in details.

> Many thanks for this comment.
> The Materials and Methods section now includes a better explanation of what modularity is and the algorithm used.
> In fact, in the original version of the essay we provided some description but we decided to remove it for the first R&R, so we are sympathetic to the comment of the reviewer. We now included a description in some detail of how the unsupervised method works. Nevertheless, we decided not to present the mathematical and algorithmic aspects in excessive detail because we regard the method as standard in the literature, and we do not want the readers to get stuck in unnecessary details.

4. Discussion: From the beginning it is unclear to me how to define 'Antivaxxers'? I searched the paper but could not find a proper definition (it should pop out immediately).

Question: Is the population of a country with a low vaccination rate (e.g. some African countries) in the category Antivaxxers? I is unclear to me if this is a negative term or neutral and if one has a choice to be in this group or is this possible determined by a third party.

In this context, it would be good to add some English-speaking African countries with a low vaccination rate and low death rate which I think exist. If the focus is only on the US a discussion of this would be sufficient.

Considering this the distinction Health-Antivax seem incorrect. I would suggest a revision of wording to make the presentation more factual.

Similar to the point about Health-Antivax also, Democrat-Republican is not well defined. Definitions of all 4 terms should be added to the methods section.

We thank the reviewer for asking us to more carefully define each group, and antivaxxers in particular. To avoid confusion, we have added a definition of 'Antivaxxers' to the introduction, specifying that we use this term in a descriptive rather than normative sense: it is our interpretation of a community of users whose top hashtags and accounts display anti-vaccination attitudes. We also note that whether or not users belong to this cluster is not contingent on their geographic location; for as explained in the Network construction and community clustering section, community membership is determined by social interaction (e.g., retweeting).

The distinctions between Democrats and Republicans on the one hand and Antivaxxers and Public Health organizations on the other are addressed in more detail in the 'community characterization and classification' section. We emphasize that since we are dealing with large and diverse populations of users about whom we only have limited information, our definitions are informed by broad patterns of similarities and differences within and between the various communities.

With respect to the U.S focused nature of our analysis, we have added a section to clarify that we *did not* limit data collection to the United States, and that the prevalence of American politics within our analysis is an artifact of the interaction between our methodology and the distribution of Twitter's English-speaking user-base.

Finally, with respect to the reviewers request to add English-speaking African countries, we note that these are already included in the original paper. In particular, our analysis of the unorthodox community makes it clear that there are African-based users involved in the discourse, and that these users express both pro- and anti-vaccination attitudes.

5. From line 407 to end: I find the connection between the numerical results and the provided discussion unsatisfactory. The main problem is a lack of connection. The authors do not use the numerical results to provide a discussion but the discussion seems not well grounded at all. At least I could not see such a connection.

It is very important that the discussion focuses exclusively on the interpretation of the numerical results. At the end of the discussion, a wider perspective, which could be even speculative, could be provided. I suggest to rewrite the entire discussion section.

The discussion section is now reorganized and rewritten in light of the reviewer's comments.

Two notes on this.
First, we sympathize with the reviewer's objection that some of what we said there was not transparently tied with the observational results that we presented before. We now organize the material in a way that is justified by the results. In particular, our discussion of the values-first vs trust-first dynamics is warranted by the observations we did in our linguistic analysis, in particular the use of moral language by different communities.
Second, the purpose of the discussion section is to provide a broader theoretical background in order to explain the observations made. For this reason, that section builds on and discusses some contemporary literature.

6. Our corpus is also mostly in the English language, which we estimate represents 68% of Twitter chatter worldwide pertaining to COVID. How can this information be relevant when Democrat-Republican are limited to the US only?

We now clarify this important point in the essay.
The prevalence of U.S-based users within our analysis is partly due to our focus on English-language discourse; partly due to the fact that the vast majority of Twitter's English speaking user-base is located in the United States (68%); and partly a reflection of the extent to which American discourse defines global online discussions, at least when those discussions are carried out in English. This explains the brute fact that random sampling using English words will most likely give an over-representation of USA discourse and dynamics, which is what we found.

Reviewer #6:

This paper presents an original and as of yet unpublished study. The authors have clearly and effectively described their analyses and drawn appropriate (and quite interesting) conclusions from the analyses presented. The article is also well written and understandable, with one exception which stood out to me while reading the manuscript.

The dataset obtained from the Twitter Streaming API appears to be global, with no mention of any geographic restrictions from users, and several non-US users are listed, with the particular grouping of the "unorthodox" being non-American in nature. However, two of the communities are defined in terms of American political divisions, the public health measure is US-centered, and geolocation data are mentioned in the discussion section when addressing future work. I think it may be helpful to clarify briefly but explicitly either in the data collection subsection or in the community characterization subsection that, even though the labels "Republican" and "Democrats" are used for two communities, the data extend globally. As a secondary, optional, matter I would be interested to know why American political cleavages may define a global discussion space.

We thank the reviewer for raising this point, and have clarified that despite identifying two clearly U.S. based clusters of users, our data extend globally. This point is made briefly in the introduction, and then elaborated in the community characterization and classification section. We note that since we did not limit data collection to the U.S. nor any other territory, the prevalence of American politics within our analysis is partly due to our focus on English-language discourse; partly due to the fact that the vast majority of Twitters English speaking user-base is located in the United States; and partly a reflection of the extent to which American political cleavages define global online discussions, at least when those discussions are carried out in English. Hence, even though we distinguish 'Democratic' and 'Republican' clusters of users, it is not the case that all users in our data set are based in the United States, for neither Public Health Institutions nor Antivaxxers are unique to U.S. discourse.

Reviewer #7:

This paper reports an interesting analysis of the discourse on vaccines around the onset of the COVID-19 pandemic. In particular, it shows how the discourse changed after the WHO officially declared the pandemic in March 2021. Overall, I think that it is well done and makes a significant contribution.

Although I review the paper for the first time, I see that it is a resubmission. Therefore, I limit my critical comments to points that can be reasonably addressed at this stage of the review process.

We thank the reviewer for their comments.

First, I encourage the authors to clarify the geographic scope of the analysis. The dataset includes only tweets written in English, but is not restricted to particular countries. At the same time, the analysis relies on the categories of US politics, particularly the distinction between Republicans and Democrats. This tension should be clarified as early as possible in the paper.

We thank the reviewer for this remark, and have added a section to the introduction to clarify the geographic scope of our analysis. This section also addresses the apparent tension between finding two distinctly U.S. based clusters and the global nature of our data set. In particular, we note that since we did not limit data collection to the U.S. nor any other territory, the prevalence of American politics within our analysis is partly due to our focus on English-language discourse; partly due to the fact that the vast majority of Twitter's English speaking user-base is located in the United States; and partly a reflection of the extent to which American political cleavages define global online discussions, at least when those discussions are carried out in English. Hence, even though we distinguish 'Democratic' and 'Republican' clusters of users, it is not the case that all users in our data set are based in the United States, for neither Public Health Institutions nor Antivaxxers are unique to U.S. discourse.

Second, the link between vaccines and COVID was a bit confusing to me, given the time frame of the analysis (December 2019-June 2020). In that period, the emphasis was not on COVID vaccines, but more on the nature of the disease and mitigation strategies such as masks and containment. The authors do frame the study as an analysis of changes of the vaccination discourse following the emergence of COVID, but they could be more explicit that the discourse is on vaccines _in general_. Currently, readers may have the wrong expectation that the paper is about COVID vaccines.

We thank the reviewer for this comment, it is important that the scope and purpose of the paper is clearly understood.

In order to clarify that the scope of the paper is how vaccine discourse *in general* changed due to Covid, we made modifications both to the Abstract and the Introduction, so that it becomes clear from the beginning.

Third, the pre-post comparisons are the most interesting parts of the analysis, but they could be discussed more in depth. For example, we see in Figure 1 that the structure of the retweet network changes significantly, but the point is barely elaborated in the text.

There are two central points about the before and after networks that are emphasized in the text now. First, that even before the pandemic communities already exhibited some polarization. Second, that engagement increased substantially after the pandemic declaration. We develop the second point in more detail through the study, in Table 2, of the ratios and total numbers of retweet behavior.

Finally, Figures 5 and 7 are not legible, partly because of the low resolution but also due to the lack of a legend and the small size of the points. These figures should be improved.

We now increased the resolution (dpi) of both figures, increased the font and point sizes in order to make those figures more legible.
